# Veratrum Viride

**Published:** 1857-08

**Authors:** 


*Veratrum Viride.*—Prof. McGugin in an article in the Iowa Medical Journal, after some considerable experience with veratrum viride, thus speaks of it: "My opinion is, that although it is a remedy which must be exhibited with care, judgment and circumspection, yet it may prove to be as certain in its own peculiar efficacy and powers as quinine, upon the powers of which, we rely with confidence, as opium in its capacity of procuring sleep, or as chloroform in the production of anesthesia. And I confidently believe that, in the future, it will be relied on with the same certainty, and with as much confidence in its powers, as an arterial sedative, as we now do, when we enter upon the exhibition of either of the above, in those cases demanding their exhibition."

